# Supplementary material for: Systematic review and meta-analysis comparing Adjustable Transobturator Male System (ATOMS) and Adjustable Continence Therapy (ProACT) for male stress incontinence
Source: PLoS One. 2019 Dec 2;14(12):e0225762. doi: 10.1371/journal.pone.0225762 (PMC6886794; doi:10.1371/journal.pone.0225762)
Supplement: S2 Table — (DOCX) [file pone.0225762.s002.docx]

**S2 Table**. Assessment of the risk of publication bias by means of Egger’s linear regression test for the study outcomes evaluated.

|  | **Slope** | **Bias** | **t** | **df** | **p-value** |
| --- | --- | --- | --- | --- | --- |
| **Dryness rate (%)** | .86 | .5 | 1.3 | 36 | .7 |
| **Improvement rate (%)** | .94 | 3.37 | 2.36 | 36 | .024 |
| **Satisfaction rate (%)** | 1.1 | -.9 | -.4 | 14 | .698 |
| **Baseline pad-count** | 3.97 | 3.93 | 1.86 | 30 | .072 |
| **Adjustment pad-count** | .95 | 2.92 | 2.52 | 28 | .018 |
| **Differential pad-count** | -3.23 | -.62 | -.29 | 28 | .775 |
| **Baseline pad-test** | 365.1 | 2.62 | 1.58 | 16 | .134 |
| **Adjustment pad-test** | 8 | 2.88 | 1.68 | 13 | .11 |
| **Differential pad-test** | -368.3 | -.01 | 1.71 | 13 | .994 |
| **Number of fillings** | 3.03 | -.67 | -.39 | 28 | .696 |
| **Complication rate (%)** | .32 | 2.58 | 1.84 | 26 | .077 |
| **Major complication rate (%)** | .11 | 2.63 | 1.84 | 10 | .096 |
| **Previous surgery for incontinence (%)** | .09 | 4.49 | 3.54 | 21 | .002 |
| **Age** | 70.34 | -.45 | -.39 | 33 | .7 |
| **Explant rate (%)** | .45 | -.95 | -.69 | 35 | .495 |
| **Months of follow-up** | 11.04 | 14.76 | 2.94 | 34 | .006 |
| **1-year durability (%)** | 1.38 | -4.27 | -.95 | 2 | .441 |
| **2-years durability (%)** | 1.24 | -3.56 | -.71 | 2 | .552 |
| **3-years durability (%)** | 1.14 | -2.15 | -.56 | 2 | .634 |
