# Supplementary material for: Systematic review and meta-analysis comparing Adjustable Transobturator Male System (ATOMS) and Adjustable Continence Therapy (ProACT) for male stress incontinence
Source: PLoS One. 2019 Dec 2;14(12):e0225762. doi: 10.1371/journal.pone.0225762 (PMC6886794; doi:10.1371/journal.pone.0225762)
Supplement: S3 Table — (DOCX) [file pone.0225762.s003.docx]

**S3 Table**. Random effect model (proportion estimate, 95% confidence Interval) and quantifying heterogeneity (I^2^, p-value) for the study outcomes evaluated.

|  | **Number of studies** | **Estimate** | **95% CI** | **heterogeneity** | |
| --- | --- | --- | --- | --- | --- |
|  |  |  |  | **I2** | **p-value** |
| **Dryness rate (%)** | 38 | 61.4 | 55.8; 66.9 | 88.4% | <.0001 |
| **Improvement rate (%)** | 38 | 85.4 | 80.4; 89.8 | 90.7% | <.0001 |
| **Satisfaction rate (%)** | 16 | 74.8 | 63.6; 84.6 | 93.5% | <.0001 |
| **Baseline pad-count** | 32 | 4.9 | 4.6; 5.2 | 99% | <.0001 |
| **Adjustment pad-count** | 30 | 1.45 | 1.3; 1.6 | 96.9% | <.0001 |
| **Differential pad-count** | 30 | -3.4 | -3.8; -3 | 98.8% | <.0001 |
| **Baseline pad-test** | 18 | 436.1 | 401.3; 470.9 | 97.1% | <.0001 |
| **Adjustment pad-test** | 15 | 34.7 | 24.9; 44.9 | 96% | <.0001 |
| **Differential pad-test** | 15 | -369.4 | -406; -332.7 | 96% | <.0001 |
| **Number of fillings** | 30 | 2.9 | 2.6; 3.2 | 97.9% | .001 |
| **Complication rate (%)** | 28 | 21 | 16.6; 25.7 | 86.2% | <.0001 |
| **Major complication rate (%)** | 12 | 5.9 | 3.1; 9.5 | 78.8% | <.0001 |
| **Previous surgery for incontinence (%)** | 23 | 14.5 | 9.7; 20.1 | 87.6% | <.0001 |
| **Age** | 35 | 69.9 | 69.3; 70.4 | 96.1% | <.0001 |
| **Explant rate (%)** | 37 | 13.3 | 9.2; 17.9 | 89.8% | <.0001 |
| **Months of follow-up** | 36 | 25.7 | 22.7; 28.7 | 99.9% | <.0001 |
| **1-year durability (%)** | 4 | 85.5 | 74.6; 0.94 | 89.7% | <.0001 |
| **2-years durability (%)** | 4 | 74.6 | 58.9; 87.7 | 90.6% | <.0001 |
| **3-years durability (%)** | 4 | 71.3 | 56.3; 84.4 | 84.1% | <.0001 |
