# Supplementary figures and images for: Systematic review and meta-analysis comparing Adjustable Transobturator Male System (ATOMS) and Adjustable Continence Therapy (ProACT) for male stress incontinence
Source: PLoS One. 2019 Dec 2;14(12):e0225762. doi: 10.1371/journal.pone.0225762 (PMC6886794; doi:10.1371/journal.pone.0225762)

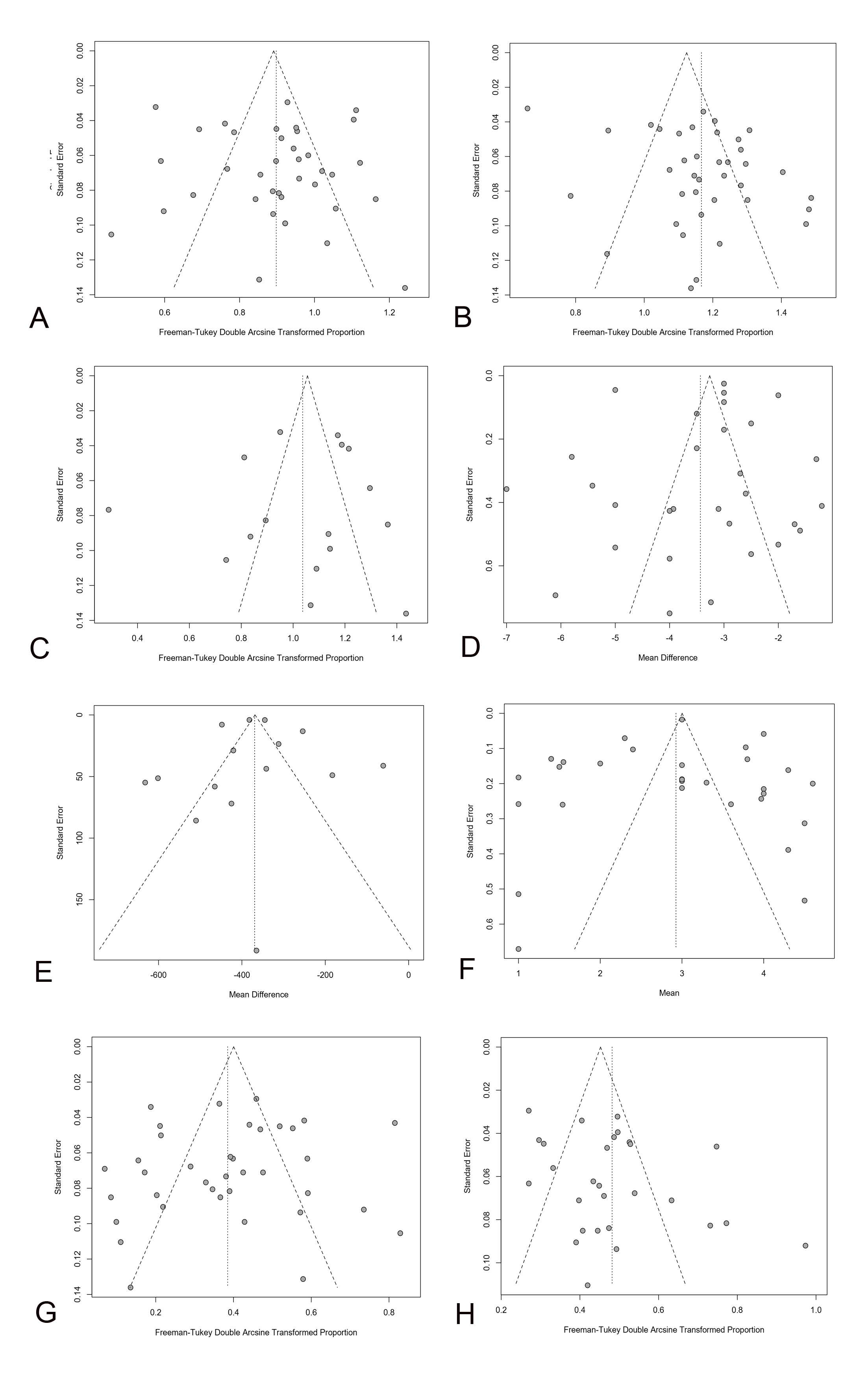

Supplement: S1 Fig — Freeman-Tukey double arcsine transform proportion for dryness (A), improvement (B), satisfaction (C), differential pad-count (D), differential pad-test (E), number of fillings (F), explant (G) and complication (H). (TIF) [file pone.0225762.s004.tif]
