# Supplementary material for: Systematic review and meta-analysis comparing Adjustable Transobturator Male System (ATOMS) and Adjustable Continence Therapy (ProACT) for male stress incontinence
Source: PLoS One. 2019 Dec 2;14(12):e0225762. doi: 10.1371/journal.pone.0225762 (PMC6886794; doi:10.1371/journal.pone.0225762)
Supplement: Attachment — Submitted filename: Response to reviewers and editor.docx [file pone.0225762.s005.docx]

**PONE-D-19-26165**

**Systematic review and meta-analysis comparing Adjustable Transobturator Male System (ATOMS) and Adjustable Continence Therapy (ProACT) for male stress incontinence.**

Dear Editors,

First we would like to thank reviewers and academic editors of PLOS ONE for their effort and the recognition of our work. We are very happy to resubmit our improved work after considering all the recommendations given.

This letter ('Response to Reviewers') is uploaded as separate file, together with a marked-up copy of the manuscript highlighting changes made ('Revised Manuscript with Track Changes').

The manuscript meets PLOS ONE's style requirements.

If the manuscript is finally accepted, I would like to make the peer review history publicly available.

**RESPONSE TO EDITOR COMMENTS**

1. As recommended the protocol has been deposited in protocols.io, and the DOI identifier assigned is: dx.doi.org/10.17504/protocols.io.8x9hxr6
2. As solicited by the Editor the label 'previous surgery' in the table is better specified as proportion of patients with previous surgery for incontinence.
3. The search strategy is specified in lines 102-103 in Material and Methods section. We confirmed we did not miss publications by using brand names or the combination of words "urinary incontinence".
4. We controlled consecutive publications from the same groups to avoid overlapping of patients in different studies. Whenever repetition was detected we only used the last publication with larger number of patients. This concept is important and is now stated in Material and Methods section (lines 115-118).
5. As recommended the discussion and conclusion sections have been limited more to what we found in your analysis. The sentence on comments about future research in the Conclusion section has been omitted. Similarly, in the Discussion section the sentences on the 'technical' differences between the devices has been omitted.
6. The sentence in the Discussion '...current evidence is still low' is made more specific and instead the overall risk of bias is stated as all the studies available are retrospective or quasi prospective single center uncontrolled cohorts (lines 272-274).
7. The editor noticed some minor occurrence(s) of overlapping text with the following previous publication of our group. A meta-analysis based of the outcomes of ATOMS alone, published in Advances and Therapy (<https://doi.org/10.1007/s12325-018-0852-4>), reference 53. This and another meta-analysis on ProACT (reference 54) have been cited and discussed form the initial writing of this new article. We also paid great attention to avoid any duplicated text outside the Methods section.
8. As requested, we have included additional details in Methods section regarding an analysis of manuscript quality assessment using the Newcastle-Ottawa scale, lines 179-185 and new Table 3.
9. While revising the submission, figure files have been uploaded to the Preflight Analysis and Conversion Engine (PACE) digital diagnostic tool to ensure that figures meet PLOS requirements.

**RESPONSE TO REVIEWERS**

**Reviewer #1:**

Comment # 1. The conclusion was that ATOMS has better efficacy and a lower complication and explantation rate. It must however be noted that the ATOMS studies had a shorter follow-up than the ProACT studies (20.8 vs 30.6 months on average).

Response to Comment #1: This important detail is completed in the conclusion section (lines 330-332).

Comment # 2: The severity of complications was not described, only the complication rate. ProACT placement is a less invasive procedure than ATOMS placement. This is summarily hinted in the Discussion, but not described quantitatively.

Response to Comment #2: The reviewer is right to state that this subject has not been properly addressed in the original version. In this revised version not only complications rate is described, but we have also reviewed and compared the proportion of major complications reported. That is lower in ATOMS than in ProACT, but again this difference did not reach statistical significance. Thereof we cannot conclude that ProACT is a less invasive procedure than ATOMS as described and this new idea is included in the revised version of the manuscript (lines 256-257, lines 302-303).

Comment # 3. There are some typing errors throughout the manuscript.

Response to Comment #3: Some typing errors have been corrected.

Comment # 4. Results. The authors write: The main etiology of incontinence was prostatectomy but other causes were included. I assume they mean radical prostatectomy (RP) and not Millin or TURP. Can the authors report the percentages of men with RP in both the ATOMS and ProACT group? Are they comparable?

Response to Comment #4: Thank you very much for this comment. The reviewer is right as we mean radical prostatectomy when we say prostatectomy. We have corrected that in the manuscript. It would be very interesting to compare the efficacy of devices not only in radical prostatectomy, but also un simple prostatectomy (Millin procedure) or transurethral resection of the prostate (TUR-P). However, that is not possible because most of the articles do not properly mention the proportion of cases derived from one or the other intervention, especially in the ProACT cohorts.

Comment # 5. The authors write NA in Table 2. This probably means Not Applicable, but I think NR (Not Reported) would be better.

Response to Comment # 5. NA is changed by NR (Not Reported) in Table 2.

**Reviewer #2:**

Comment #1. Minor spelling mistakes in the paper can easily be fixed.

Response to Comment #1: Some spelling mistakes ad minor typing errors have been corrected.

Comment # 2. Not only the outcome parameters, but also patient characteristics would be compared. All studies have provided statistics concerning their patients like age and type of prior operation. Here the authors should show, that there are no significant differences between ProAct and Atoms groups, since this would make the conclusion stronger.

Response to Comment #2: Thank you very much for this comment. The reviewer is right to ask not only to compare outcomes of the different devices, but also similarity between characteristics of the patients treated by one or the other device. Baseline pad-count (the most frequent objective measurement in most publications) is equivalent between cohorts. Patient age is included in new Table 2 for each study. Type of previous incontinence operation is not available for each cohort in the publications, as a variety of options are possible; however, we have analysed the proportion of patients with previous anti-incontinence operation. New pooled analysis of patient age at the time of implant, baseline pad-count and the proportion of patients previously intervened with both techniques are similar in the literature, what confirms cohorts are apt to be compared. As suggested by the reviewer these data are included both in Results section (lines 205-213) and in revised Supplementary material S3 and S4.
